# Correction: Cheng et al. Advances in the Biosynthesis of Plant Terpenoids: Models, Mechanisms, and Applications. *Plants* 2025, *14*, 1428

**DOI:** 10.3390/plants15091336

**Published:** 2026-04-28

**Authors:** Renwu Cheng, Shuqi Yang, Dongli Wang, Fangcuo Qin, Shengkun Wang, Sen Meng

**Affiliations:** 1 Guangzhou Collaborative Innovation Center on Science-Tech of Ecology and Landscape, Guangzhou 510520, China; 2 State Key Laboratory of Tree Genetics and Breeding, Research Institute of Tropical Forestry, Chinese Academy of Forestry, Guangzhou 510520, China; 3 College of Agriculture, Guangxi University, Nanning 530004, China; 4 Jiangxi Provincial Key Laboratory of Plantation and High Valued Utilization of Specialty Fruit Tree and Tea, Institute of Biological Resources, Jiangxi Academy of Sciences, Nanchang 330096, China

## Missing Citation

The original publication [1] did not include the following citation: “Karunanithi, P.S.; Philipp, Z. Terpene synthases as metabolic gatekeepers in the evolution of plant terpenoid chemical diversity. *Front. Plant Sci.* **2019**, *10*, 1166”. This reference has now been inserted in the caption of Figure 4 as new ref [43] as follows:

**Figure 4.** Evolutionary relationships among TPS based on protein structures. The domain color marks the γ-domain (orange), the β-domain (blue), the α-domain (red) as well as the conserved DxDD (green) and DDx2D (cyan) motifs [43].

With this correction, the order of some references has been adjusted accordingly. The authors state that the scientific conclusions are unaffected. This correction was approved by the Academic Editor. The original publication has also been updated.
